# Prognostic utility of a multi-biomarker panel in patients with suspected myocardial infarction

**DOI:** 10.1007/s00392-023-02345-7

**Published:** 2023-12-11

**Authors:** Betül Toprak, Jessica Weimann, Jonas Lehmacher, Paul M. Haller, Tau S. Hartikainen, Alina Schock, Mahir Karakas, Thomas Renné, Tanja Zeller, Raphael Twerenbold, Nils A. Sörensen, Dirk Westermann, Johannes T. Neumann

**Affiliations:** 1 Department of Cardiology, University Heart & Vascular Center Hamburg, University Medical Center Hamburg-Eppendorf, Martinistraße 52, 20246 Hamburg, Germany; 2 German Center for Cardiovascular Research (DZHK), Partner Site Hamburg/Kiel/Lübeck, Hamburg, Germany; 3 Department of Cardiology, University Heart Center Freiburg - Bad Krozingen, Bad Krozingen, Germany; 4 Department of Intensive Care Medicine, Center for Anesthesiology and Intensive Care Medicine, University Medical Center Hamburg-Eppendorf, Hamburg, Germany; 5 Institute of Clinical Chemistry and Laboratory Medicine, University Medical Center Hamburg-Eppendorf, Hamburg, Germany; 6 Irish Centre for Vascular Biology, School of Pharmacy and Biomolecular Sciences, Royal College of Surgeons in Ireland, Dublin, Ireland; 7 Center for Thrombosis and Hemostasis (CTH), Johannes Gutenberg University Medical Center, Mainz, Germany; 8 University Center of Cardiovascular Science (UCCS), University Heart and Vascular Center Hamburg, Hamburg, Germany; 9 Department of Epidemiology and Preventive Medicine, School of Public Health and Preventive Medicine, Monash University, Melbourne, Australia

**Keywords:** Risk prediction, Acute coronary syndrome, Chest pain, Biomarker, NT-proBNP

## Abstract

**Background:**

The accurate identification of patients with high cardiovascular risk in suspected myocardial infarction (MI) is an unmet clinical need. Therefore, we sought to investigate the prognostic utility of a multi-biomarker panel with 29 different biomarkers in in 748 consecutive patients with symptoms indicative of MI using a machine learning-based approach.

**Methods:**

Incident major cardiovascular events (MACE) were documented within 1 year after the index admission. The selection of the best multi-biomarker model was performed using the least absolute shrinkage and selection operator (LASSO). The independent and additive utility of selected biomarkers was compared to a clinical reference model and the Global Registry of Acute Coronary Events (GRACE) Score, respectively. Findings were validated using internal cross-validation.

**Results:**

Median age of the study population was 64 years. At 1 year of follow-up, 160 cases of incident MACE were documented. 16 of the investigated 29 biomarkers were significantly associated with 1-year MACE. Three biomarkers including NT-proBNP (HR per SD 1.24), Apolipoprotein A-I (Apo A-I; HR per SD 0.98) and kidney injury molecule-1 (KIM-1; HR per SD 1.06) were identified as independent predictors of 1-year MACE. Although the discriminative ability of the selected multi-biomarker model was rather moderate, the addition of these biomarkers to the clinical reference model and the GRACE score improved model performances markedly (∆C-index 0.047 and 0.04, respectively).

**Conclusion:**

NT-proBNP, Apo A-I and KIM-1 emerged as strongest independent predictors of 1-year MACE in patients with suspected MI. Their integration into clinical risk prediction models may improve personalized risk stratification.

**Graphical abstract:**

Prognostic utility of a multi-biomarker approach in suspected myocardial infarction. In a cohort of 748 patients with symptoms indicative of myocardial infarction (MI) to the emergency department, we measured a 29-biomarker panel and performed regressions, machine learning (ML)-based variable selection and discriminative/reclassification analyses. We identified three biomarkers as top predictors for 1-year major adverse cardiovascular events (MACE). Their integration into a clinical risk prediction model and the Global Registry of Acute Coronary Events (GRACE) Score allowed for marked improvement in discrimination and reclassification for 1-year MACE. *Apo* apolipoprotein; *CRP* C-reactive protein; *CRS* clinical risk score; *ECG* electrocardiogram; *EN-RAGE* extracellular newly identified receptor for advanced glycation end-products binding protein; *FABP* fatty acid–binding protein; *GS* Grace Score; *hs-cTnI* high-sensitivity cardiac troponin I; *KIM-1* kidney injury molecule–1; *LASSO* least absolute shrinkage and selection operator; *MACE* major adverse cardiovascular events; *MI* myocardial infarction; *NRI* net reclassification improvement; *NT-proBNP* N-terminal prohormone of brain natriuretic peptide.

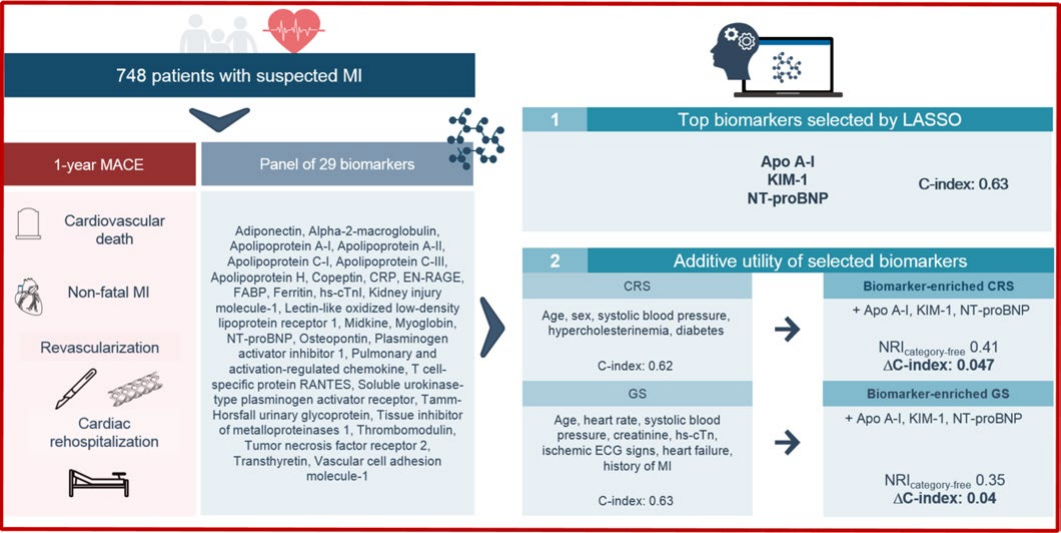

**Supplementary Information:**

The online version contains supplementary material available at 10.1007/s00392-023-02345-7.

## Introduction

Globally, suspected myocardial infarction (MI) remains a major reason for presentation to the emergency departments (ED) [1, 2]. There are well-established algorithms to diagnose or rule-out MI [3–5]. Since their introduction, high-sensitivity cardiac troponin (hs-cTn) assays have dictated the diagnostic workup in chest pain patients [6–8]. In this setting, no other circulating biomarker was proven to exhibit nearly as much utility as hs-cTn to date [4, 9].

Even after the exclusion of an acute MI, further investigations may be warranted in some patients who are at a high risk of major adverse cardiovascular events (MACE) [10, 11]. Beyond clinical risk factors, circulating biomarkers may provide incremental prognostic information by mirroring major pathophysiological pathways, in turn, potentially indicating cardiovascular sequelae [8]. Novel integrative approaches combining multiple biomarkers which reflect complimentary and multifaceted biological processes might be particularly useful for prognostic purposes. Previously, a multi-biomarker-based score consisting of four circulating biomarkers (N-terminal fragment of brain natriuretic peptide prohormone [NT-proBNP], osteopontin, kidney injury molecule-1 [KIM-1] and tissue inhibitor of metalloproteinases 1 [TIMP-1]) was derived and validated in patients who underwent coronary and peripheral angiography. However, this score was developed for application in an intermediate-to high-risk patient collective [11].

Comparable predictive models for unselected chest pain populations represent an unmet clinical need as the identification of at-risk individuals remains particularly challenging in a heterogeneous, ‘all-comers’ ED setting [2].

Therefore, we aimed to investigate the prognostic utility of a multi-biomarker panel with 29 different biomarkers mirroring different pathophysiological pathways in a contemporary cohort of patients presenting to the ED with suspected MI.

## Methods

### Study population and adjudication

We used data from the Biomarkers in Acute Cardiac Care (BACC) study (NCT02355457, ClinicalTrials.gov), which was approved by the local ethics committee Hamburg (Ethikkomission der Ärztekammer). All study participants provided written informed consent.

BACC is an ongoing prospective cohort study investigating patients aged 18 years or older who present to the ED with symptoms suggestive of MI. All patients included in our analyses were prospectively enrolled at the ED of the University Medical Center Hamburg-Eppendorf between July 2013 and November 2017. These patients underwent a standardized diagnostic work-up (collection of clinical data, electrocardiogram [ECG], vital parameters, and blood samples) at admission with subsequent guideline-based management. Patients with ST-segment elevation MI were excluded from further analyses.

The final diagnosis of each participant was adjudicated according to the fourth Universal Definition of MI [12] by two physicians in a blinded fashion. A third physician was consulted only in case of disagreement. Available clinical, imaging and laboratory parameters including hs-cTnT (Elecsys, Roche Diagnostics^®^) with respective sex-specific cut-off values (9 ng/L for women, 15.5 ng/L for men) formed the basis for adjudication.

### Biomarker measurements

The multi-biomarker panel including 29 biomarkers was measured in blood samples which were collected directly at admission of the index presentation to the ED and thereafter stored at − 80 °C following a standardized protocol.

Overall, 4 out of 29 biomarkers of the panel were measured using different assays: hs-cTnI—the Architect i1000SR immunoassay, Abbott Diagnostics^®^; copeptin—Brahms copeptin ultrasensitive immunoluminometric assay on the Kryptor Compact Plus System, Thermo Fisher Scientific^®^; soluble urokinase-type plasminogen activator receptor (suPAR)—suPARnostic standard enzyme-linked immunosorbent assay, ViroGates^®^; C-reactive protein (CRP)—Siemens Dimension Vista analyzer, Siemens Healthineers^®^. All other 25 biomarkers were jointly measured on the Luminex xMAP Platform, Luminex^®^, which is a multiplexed, microsphere-based assay system [10, 13].

These included adiponectin, alpha-2-macroglobulin (A2Macro), apolipoprotein (Apo) A-I, Apo A-II, Apo C-I, Apo C-III, Apo H, extracellular newly identified receptor for advanced glycation end-products binding protein (EN-RAGE), fatty acid–binding protein (FABP), ferritin, KIM-1, lectin-like oxidized low-density lipoprotein receptor 1 (LOX-1), midkine, myoglobin, NT-proBNP, osteopontin, plasminogen activator inhibitor 1 (PAI-1), pulmonary and activation-regulated chemokine (PARC), T cell–specific protein regulated upon activation, normal T cell expressed and presumably secreted (RANTES), Tamm–Horsfall urinary glycoprotein (THP), TIMP-1, thrombomodulin (TM), tumor necrosis factor receptor 2 (TNFR2), transthyretin (TTR), and vascular cell adhesion molecule–1 (VCAM-1).

### Statistical analyses

Baseline characteristics are provided as absolute (and relative) frequencies for categorical variables and as median (25th, 75th percentile) for continuous variables. To achieve a near-normal distribution, all investigated biomarkers were log-transformed for the analyses. Major adverse cardiovascular events (MACE) were defined as the composite of cardiovascular death, non-fatal MI (excluding index events), revascularization, and cardiac rehospitalization within 1 year after admission to the ED.

First, all biomarkers were investigated in (a) an unadjusted, and (b) age-and sex-adjusted, univariate Cox regression model to calculate the respective hazard ratio (HR) per standard deviation (SD) for 1-year MACE. Firth penalization was used for these analyses to minder the problem of overfitting.

Second, the best multi-biomarker model for 1-year MACE was selected using Least Absolute Shrinkage and Selection Operator (LASSO, in package glmnet), a machine learning (ML) technique amending traditional regression models, with fivefold cross-validation of estimators. Predictors were ranked by their respective HR per SD. The variables for adjustment, age and sex, were forced to stay in the model-building process. For the LASSO-selected model, the cumulative C-index [14] was calculated using the information on time-to-event and corrected via bootstrapping (*B* = 500). If multiple events occurred in one patient, the earliest time-to-event (shortest time range) was chosen. Further, the Akaike information criterion (AIC) was additionally computed to weigh the goodness-of-fit and the simplicity of the model, and to ensure a better inter-model comparability—the lower the AIC, the better is the quality of the model.

The discriminative ability of the LASSO-selected model was then compared to (1) a clinical reference model (including age, sex, systolic blood pressure, hyperlipoproteinemia, current smoker, and diabetes) and (2) the Global Registry of Acute Coronary Events (GRACE) score [15]. To assess the additional predictive value of the biomarkers as identified by LASSO selection when compared to the clinical reference model and the GRACE score, the category-free net reclassification index (NRI, values ranging between − 2 and 2) using the Kaplan–Meier method as suggested by Pencina and colleagues [16] was calculated and validated by fivefold cross-validation, respectively. A high NRI for cases (non-cases) indicates that the addition of selected biomarkers to the reference model is particularly helpful for identifying individuals at a high risk (at a low risk) of 1-year MACE. Lastly, Kaplan–Meier curves for freedom from MACE within 1 year after admission were computed by quartiles of each LASSO-selected biomarkers. To test for any differences between the quartiles the log-rank test was applied.

A *p* value of < 0.05 was considered statistically significant. All statistical analyses were performed using R statistical version 4.0.3 (http://www.R-project.org).

## Results

### Baseline characteristics

The multi-biomarker panel was measured in 748 patients with a median age at admission of 64 (interquartile range [IQR] 50–75) years, 472 (63.1%) were men. In the overall study population, 490 (65.9%) patients had hypertension, 277 (37.0%) had hyperlipoproteinemia, 94 (12.7%) had diabetes, and 202 (27.1%) were current smokers. Among all participants, 138 (18.5%) were diagnosed with MI, of whom 107 (14.3%) were classified as having type 1 MI and 31 (4.2%) as type 2 MI, and 221 (29.6%) with myocardial injury (Table 1). Log-transformed baseline concentrations of all 29 biomarkers as part of the panel are presented in Online Resource 1.

**Table 1:** Baseline characteristics of the study population

	All (*N* = 748)
Age (years)	64.0 (50.0, 75.0)
Male, no. (%)	472 (63.1)
BMI (kg/m^2^)	26.0 (23.4, 29.0)
Hypertension, no. (%)	490 (65.9)
Systolic blood pressure (mmHg)	146.0 (130.0, 161.0)
Diastolic blood pressure (mmHg)	83.0 (74.8, 91.0)
Hyperlipoproteinemia, no. (%)	277 (37.0)
Diabetes, no. (%)	94 (12.7)
Current smoker, no. (%)	202 (27.1)
Family history of CAD, vo. (%)	132 (18.4)
History of CAD, no. (%)	240 (32.1)
Ischemic signs on baseline ECG, No. (%)	160 (22.2)
Symptom onset time, no. (%)	
< 1 h	51 (7.2)
1 to < 3 h	158 (22.4)
3 to < 6 h	79 (11.2)
6 to < 12 h	91 (12.9)
≥ 12 h	326 (46.2)
Diagnosis according to fourth UDMI, No. (%)	
MI	138 (18.5)
Type 1 MI	107 (14.3)
Type 2 MI	31 (4.2)
Myocardial injury	221 (29.6)
Non-MI	387 (51.9)
GRACE score (points)	99.0 (68.0, 128.0)

Values are presented as median (interquartile range) for continuous variables or No. (%) for binary variables

*BMI* Body mass index; *CAD* coronary artery disease; *ECG* electrocardiogram; *GRACE* global registry of acute coronary events; *MI* myocardial infarction; *PCI* percutaneous coronary intervention; *UDMI* universal definition of myocardial infarction

### Predictive utility of single biomarkers for 1-year MACE

**Fig. 1:**
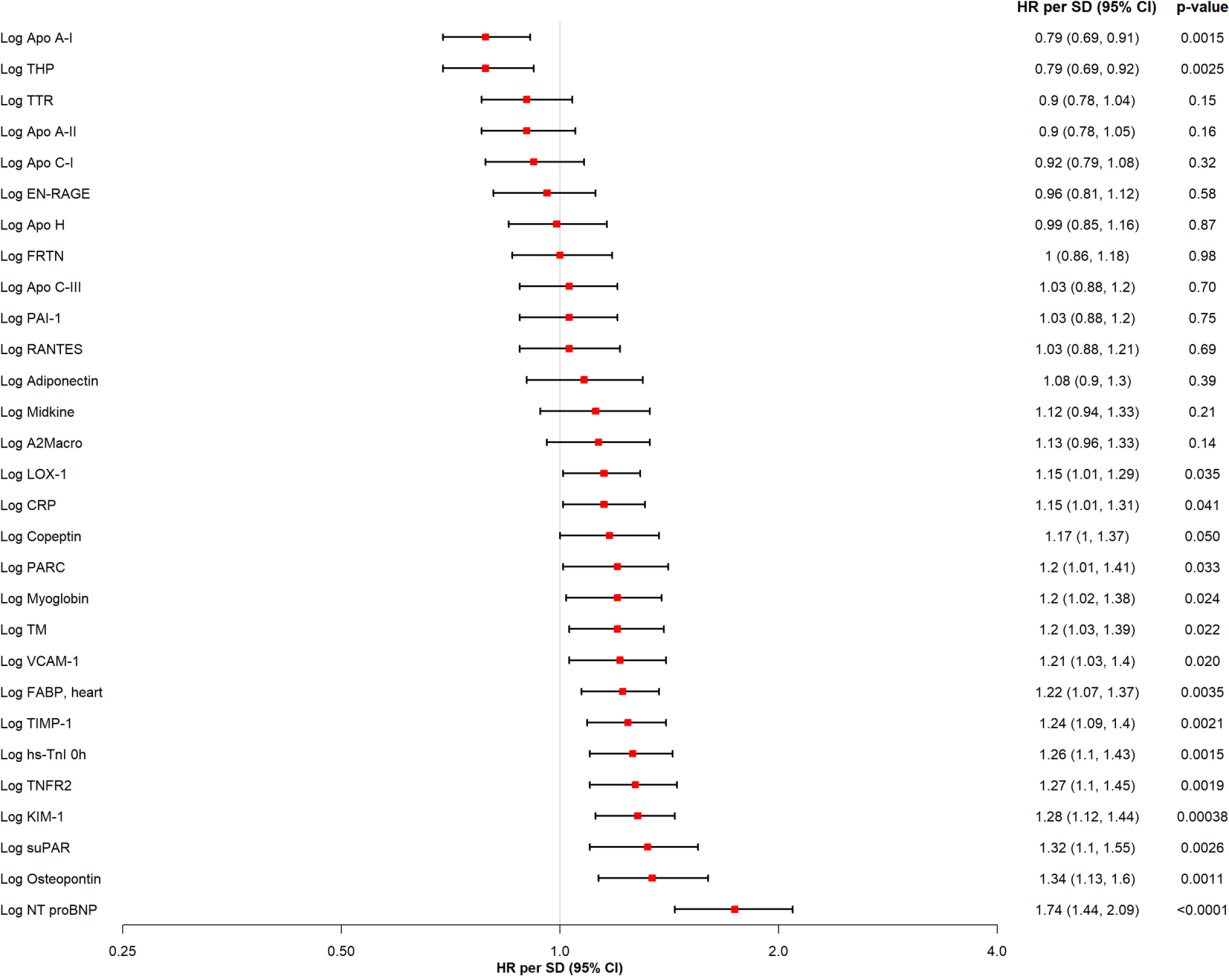
Univariable Cox Regression Analysis for the Prediction of 1-year MACE. Provided are the hazard ratios (HR) per standard deviation (SD) with respective 95% confidence intervals (CI) in ascending sequence from the top to the bottom for each biomarker in the univariable, age-/sex-adjusted Cox regression model for 1-year MACE. *A2Macro* alpha-2-macroglobulin; *Apo* apolipoprotein; *CI* confidence interval; *CRP* C-reactive protein; *EN-RAGE* extracellular newly identified receptor for advanced glycation end-products binding protein; *FABP* fatty acid–binding protein; *HR* hazard ratio; *hs-cTnI *high-sensitivity cardiac troponin I; *KIM-1* kidney injury molecule–1; *LOX-1* lectin-like oxidized low-density lipoprotein receptor 1; *NT-proBNP* N-terminal prohormone of brain natriuretic peptide; *PAI-1* plasminogen activator inhibitor 1; *PARC* pulmonary and activation-regulated chemokine; *RANTES* T cell–specific protein RANTES; *SD* standard deviation; *suPAR* soluble urokinase-type plasminogen activator receptor; *THP* Tamm-Horsfall urinary glycoprotein; *TIMP-1* tissue inhibitor ofmetalloproteinases 1; *TM* thrombomodulin; *TNFR2* tumor necrosis factor receptor 2; *TTR*  transthyretin; *VCAM-1* vascular cell adhesion molecule–1

Overall, median follow-up was 5.79 (95% CI 5.7–5.92) years after index presentation. At 1 year after admission, 160 (22.5%) participants experienced MACE (Online Resource 2). For 1-year MACE, 21 of the investigated 29 biomarkers were significantly associated with 1-year MACE in crude univariate analyses (Online Resource 3). After adjustment for age and sex, 16 biomarkers still provided significant predictive utility, of which Apo A-I (HR per SD 0.79, 95% CI 0.69–0.91, *p* = 0.0015) and THP (HR per SD 0.79, 95% CI 0.69–0.92, *p* = 0.0025) were inversely associated with 1-year MACE, while the remaining 14 biomarkers exhibited positive associations. Amongst the latter, NT-proBNP (HR per SD 1.74, 95% CI 1.44–2.09, *p* < 0.0001), osteopontin (HR per SD 1.34, 95% CI 1.13–1.6, *p* = 0.0011), suPAR (HR per SD 1.32, 95% CI 1.1–1.55, *p* = 0.0026), and KIM-1 (HR per SD 1.28, 95% CI 1.12–1.44, *p* = 0.0004) were the strongest predictors of 1-year MACE (Fig. 1).

### Multi-biomarker model for the prediction of 1-year MACE

Applying LASSO for variable selection, three biomarkers including Apo A-I (HR per SD 0.98), KIM-1 (HR per SD 1.06) and NT-proBNP (HR per SD 1.24) were identified as independent predictors of 1-year MACE, and thus were combined into a model also including age and sex (Table 2).

**Table 2:** Multi-biomarker model adjusted by age and sex for 1-year MACE selected by LASSO

	HR	HR per SD
Log Apo A-I	0.94	0.98
Log KIM-1	1.11	1.06
Log NT proBNP	1.13	1.24
Performance of selected multivariable model		
Uncorrected C-index	0.65	
Corrected C-index	0.63	
AIC	1862.46	

The best multi-biomarker model for 1-year MACE was selected using LASSO with fivefold cross-validation of estimators. Predictors were ranked by their respective hazard ratio (HR) per standard deviation (SD). The variables for adjustment, age and sex, were forced to stay in the model. For the LASSO-selected model, the C-index (corrected for over-optimism) was calculated and the Akaike information criterion (AIC) was computed

*AIC* Akaike information criterion; *Apo* apolipoprotein; *HR* hazard ratio; *KIM-1* kindey injury molecule-1; *NT-proBNP* N-terminal prohormone of brain natriuretic peptide; *SD* standard deviation

In Kaplan–Meier analyses plotted for 1-year MACE by quartiles of selected biomarker, statistically significant differences between the quartiles were observed for Apo A-I (*p* = 0.0065) and NT-proBNP (*p* < 0.0001), but not for KIM-1 (Online Resource 5).

The LASSO-selected model yielded a moderate discriminative ability (corrected C-index 0.63, AIC 1862.46; Table 2), which, however, exceeded that of the clinical prediction model (corrected C-index 0.617, AIC 1962.7; Table 3) and comparable to the GRACE Score (corrected C-index 0.629, AIC 2025.2; Table 4). Main predictors in the clinical model were age, systolic blood pressure and the presence of diabetes (Table 3).

**Table 3:** Clinical model without and with LASSO-selected biomarkers

	Clinical model		Clinical model + selected biomarkers	
	HR	*p* value	HR	*p* value
Age (years)	1.02 (1.01, 1.03)	< 0.001	1.00 (0.99, 1.02)	0.68
Male sex	1.26 (0.89, 1.77)	0.19	1.15 (0.81, 1.64)	0.42
SBP (mmHg)	0.99 (0.99, 1.00)	0.041	1.00 (0.99, 1.00)	0.46
Hyperlipoproteinemia	1.16 (0.84, 1.61)	0.37	1.13 (0.82, 1.58)	0.45
Current smoker	0.84 (0.55, 1.28)	0.42	0.86 (0.56, 1.32)	0.49
Diabetes	1.71 (1.16, 2.53)	0.0072	1.32 (0.86, 2.02)	0.21
Log Apo A-I			0.61 (0.37, 1.00)	0.051
Log KIM-1			1.16 (0.87, 1.56)	0.31
Log NT proBNP			1.32 (1.17, 1.49)	< 0.001
Performance of multivariable models				
Uncorrected C-index	0.637		0.688	
Corrected C-index	0.617		0.664	
AIC	1962.7		1936.7	
NRI_category-free_ (95% CI)	–		0.41 (0.24, 0.60)	
NRI_cases_ (95% CI)	–		0.10 (− 0.055, 0.26)	
NRI_non-cases_ (95% CI)	–		0.31 (0.23, 0.39)	

A clinical risk model including traditional cardiovascular risk factors (including age, sex, systolic blood pressure, hyperlipoproteinemia, current smoker, and diabetes) was computed with respective hazard ratios (HR) per standard deviation (SD) of each component. Three LASSO-selected biomarkers were added to this model. The performance of this biomarker-enhanced model was compared to the clinical reference model using C-index (corrected for over-optimism), Akaike Information Criterion (AIC) and net reclassification improvement (NRI) for cases (i.e. with 1-year MACE) and non-cases (i.e. without 1-year MACE) with respective 95% confidence intervals (95% CI)

*AIC* Akaike information criterion; *Apo* apolipoprotein; *CI*  confidence interval; *HR* hazard ratio; *KIM-1* kidney injury molecule-1; *NRI* net reclassification index; *NT-proBNP* N-terminal prohormone of brain natriuretic peptide; *SBP* systolic blood pressure; *SD* standard deviation

**Table 4:** GRACE score without and with LASSO-selected biomarkers

	GRACE score		GRACE score + selected biomarkers	
	HR	*p* value	HR	*p* value
GRACE score	1.01 (1.01, 1.02)	< 0.001	1.00 (1.00, 1.01)	0.18
Log Apo A-I			0.56 (0.36, 0.88)	0.012
Log KIM-1			1.27 (0.98, 1.65)	0.070
Log NT proBNP			1.26 (1.10, 1.44)	< 0.001
Performance of multivariable models				
Uncorrected C-index	0.636		0.677	
Corrected C-index	0.629		0.669	
AIC	2025.2		2004.6	
NRI_category-free_ (95% CI)	–		0.35 (0.17, 0.52)	
NRI_cases_ (95% CI)	–		0.088 (– 0.070, 0.23)	
NRI_non-cases_ (95% CI)	–		0.26 (0.17, 0.34)	

The Global Registry of Acute Coronary Events (GRACE) Score was applied. Three LASSO-selected biomarkers were added to this model. The performance of this biomarker-enhanced model was compared to the GRACE Score using C-index (corrected for over-optimism), Akaike Information Criterion (AIC) and net reclassification improvement (NRI) for cases (i.e. with 1-year MACE) and non-cases (i.e. without 1-year MACE) with respective 95% confidence intervals (95% CI)

*AIC* Akaike information criterion; *Apo* apolipoprotein; *CI* confidence interval; *GRACE* global registry of acute coronary events; *HR* hazard ratio; *KIM-1* kidney injury molecule-1; *NRI* net reclassification index; *NT-proBNP* N-terminal prohormone of brain natriuretic peptide; *SBP* systolic blood pressure; *SD* standard deviation

### Additive predictive utility of selected biomarkers for 1-year MACE

The addition of all three LASSO-selected biomarkers to the clinical reference model (corrected C-index 0.617) yielded a marked increase regarding the discriminative ability of the resulting expanded (biomarker-enriched) clinical model by ∆C-index 0.047 with an overall NRI_category-free_ of 0.41 (95% CI 0.24–0.60), which was primarily driven by the down reclassification of 346 (66.2%) non-cases (NRI_non-cases_ 0.31, 95% CI 0.23–0.39; Table 3; Online Resource 4).

Similarly, adding the three biomarkers to the GRACE score (corrected C-index 0.629) led to a substantial improvement of discrimination by the expanded (biomarker-enriched) GRACE model by ∆C-index 0.04. The overall NRI_category-free_ was 0.35 (95% CI 0.17–0.52), which again resulted from a higher proportion of reclassifications for non-cases (NRI non-cases 0.26, 95% CI 0.17– biomarker-enriched 0.34; Table 4; Online Resource 4).

## Discussion

In this analysis of the BACC study, we investigated the prognostic utility of 29 biomarkers based on an ML-based approach and identified three of them—NT-proBNP, Apo A-I and KIM-1—as best-predictive biomarkers for 1-year MACE in an unselected cohort of patients with suspected MI. Combining these three biomarkers into a multi-biomarker model yielded slightly better or similar discriminative ability when compared to the clinical risk model and the GRACE score, respectively. However, the integration of the three LASSO-selected biomarkers into both, the clinical risk model and the GRACE score, led to a marked improvement of discrimination and reclassification for 1-year MACE in this chest pain collective (Graphic abstract). This work builds upon our previous investigation on the discriminative ability of the same multi-biomarker panel for distinguishing type 1MI, type 2 MI, and myocardial injury [13].

Each year about 20 million patients present to the ED in Europe and North America with suspicion of MI [1, 2]. However, chest pain remains a condition of heterogeneous origin and only 5 to 25% receive the final diagnosis of MI based on well-established, guideline-based diagnostic algorithms [17]. Even after the exclusion of an acute MI, some patients remain at high risk of cardiovascular events and therefore merit further diagnostic workup for individual decision-making in the ED and/or closer follow-up in the outpatient care setting [10]. The identification of such at-risk patients poses a yet unsolved challenge to clinicians in a busy, often overcrowded ED setting—risk prediction models may help to close this gap.

Circulating biomarkers serve as objective, easy to quantify blood proteins that mirror distinct pathophysiological pathways and may provide biologically derived, prognostic information beyond clinical risk factors [11]. For this purpose, several biomarkers were investigated in the past years for their predictive ability in chest pain patients [18–20]. Among established biomarkers, natriuretic peptides such as B-type natriuretic peptide and the fragment of its precursor, NT-proBNP, as indicators of myocardial stress and stretch, were identified as important circulating predictors of cardiovascular events in populations with chest pain and potential ACS in numerous previously published analyses [19, 21, 22]. Similarly, NT-proBNP ranked as the strongest predictor of 1-year MACE among 29 biomarkers investigated in our study. This finding might be, at least partly, driven by the proportion of heart failure (HF) patients with a higher risk of cardiac rehospitalization and cardiovascular death [23]. However, NT-proBNP was shown to provide prognostic information even in the absence of HF [24]. Although hs-cTn was also shown to add prognostic information in patients with suspected myocardial infarction [25], its diagnostic utility remains clearly outpacing, also in other studies [10]. Further, blood lipids are the major drivers of atherosclerotic processes and therefore targeted by primary and secondary preventive strategies [26, 27]. Beyond traditional lipid measures, the protein components of plasma lipoproteins are increasingly known to bear potential for risk prediction [28]. In line with its known anti-atherogenic effect, Apo A-I, a major component of the HDL particle, was consistently associated with a significantly lower risk of 1-year MACE in our study. Our findings are in line with previous analyses [29], and Apo A-I was also selected as one of the top predictors into our multi-biomarker model. This might be particularly striking when considering Apo A-I for potential therapeutic issues [30]. Beyond established proteins, the search for prognostic utility has been extended towards novel and emerging biomarkers in recent years. In particular, deciphering the interplay between renal axis and cardiovascular risk attracted major research interest [31]. Among 29 different biomarkers measured in our study collective, KIM-1 was selected as an important predictor of 1-year MACE in suspected myocardial infarction. Besides its role as a novel marker of kidney damage, KIM-1 has been previously identified as a prognostic marker in cardiovascular disease [11]. One might hypothesize that KIM-1 performs particularly well in identifying chest pain patients with a high risk of adverse cardiovascular events as renal impairment often reflects age and comorbidities. However, in Kaplan–Meier analyses plotted for 1-year MACE by quartiles of biomarker levels, KIM-1 did not reach statistical significance, which suggests a synergistic effect in the LASSO-selected multi-biomarker model rather than a standalone prognostic value.

In fact, as multiple pathways contribute to atherosclerotic plaque development and instability, subsequent cardiovascular sequelae, and organ dysfunctions which will ultimately impact outcome, the integration of biologic information from several biomarkers may improve prognostication [10]. Using a panel of 109 biomarkers, McCarthy and colleagues recently developed a biomarker-based risk score for the prediction of 1-year MACE in 927 patients in the Catheter Sampled Blood Archive in Cardiovascular Diseases Study (CASABLANCA). The final panel included a total of four selected biomarkers: NT-proBNP, osteopontin, KIM-1 and TIMP-1 [11]. Previously, this risk model was externally validated in BACC [10].

Despite some similarities with our findings regarding selected best-predictive biomarkers (i.e. NT-proBNP, KIM-1), which supports their potential prognostic value and might be explained by partly overlapping patient subgroups (i.e. those undergoing coronary angiography in BACC) between the two cohorts, it is important to note that the risk score provided by McCarthy et al. targets a preselected and “pre-triaged”, intermediate- to high-risk population who underwent coronary and peripheral angiography [11]. In contrast, we aimed to specifically derive a multi-biomarker model for predicting MACE in an unselected cohort of patients with symptoms suggestive of MI. This all-comers, ED collective harbors heterogeneous risk profiles and is particularly challenging to risk stratify. From a clinical perspective, identifying and discharging low-risk patients with chest pain rapidly is crucial to prevent overcrowding and enable for process optimization as well as judicious use of healthcare resources in the ED [4]. In line with this clinical rationale, the addition of LASSO-selected biomarkers to both, the clinical risk model and GRACE score, yielded a remarkable improvement regarding net reclassification in our study, which was primarily driven by the patient fraction free of MACE at 1 year after admission to the ED.

Further, the score derived in the CASABLANCA cohort is solely composed of selected biomarkers as no clinical variable was chosen during the model-selection process [11]. In BACC, the LASSO-selected multi-biomarker model only reached moderate discriminative ability with a C-index of 0.63; however, the addition of the top three predictive biomarkers to both, the clinical risk model and GRACE score, led to a substantial increase in discriminative ability. Our findings underline the importance of taking patient characteristics and clinical risk factors as major determinants for risk triage and individual decision-making into account [4].

There are various clinical risk factors and scores commonly used to mortality in selected patient groups, i.e. the GRACE score [32] integrating variables from patient history, clinical examination, ECG and laboratory testing for patients with ACS or the Thrombolysis with Myocardial Infarction (TIMI) risk score [33] estimating mortality in patients with unstable angina and non-ST elevation MI. However, besides predicting mortality and not MACE, none of these risk scores has been specifically developed to aid risk stratification in a heterogeneous collective of patients with chest discomfort and possible MI [34].

### Strengths and limitations

The major strength of our study concerns the underlying well-characterized dataset of the BACC cohort study with thoroughly adjudicated diagnoses according to the fourth Universal Definition of MI. Although the final sample size eligible for our study, i.e. with available multi-biomarker panel, was limited, the measurement of 29 different established and emerging biomarkers is a valuable and unique feature. However, we also acknowledge that the panel includes multiple biomarkers which are not measured as part of clinical routine.

In spite of having corrected for over-optimism of applying the models to the same dataset, the main limitation of this study remains the lack of external validation of derived findings.

## Conclusion and future directions

Patients presenting with symptoms suggestive of MI are heterogeneous and challenging, and may need further diagnostic workup even after the exclusion of an acute MI. Among 29 distinct biomarkers, an ML-based approach identified three top-scoring biomarkers—NT-proBNP, Apo A-I and KIM-1—for the prediction of 1-year MACE in patients with symptoms indicative of MI. Upon integration into biomarker-enriched clinical models, these markers improved discriminative ability and reclassification for 1-year MACE markedly.

Thus, biomarker-enriched prediction models could improve prognostication in the ED setting and add to the current diagnostic workup in chest pain patients. As the additional measurement of biomarkers for prognostic information was previously not recommended by the ESC guidelines [4], further investigation is needed to examine the net clinical benefit, i.e. efficacy and impact on patient outcomes, as well as feasibility, and cost-effectiveness of biomarker-enriched risk prediction models for prognostication in suspected MI in the ED setting.

## Supplementary Information

Below is the link to the electronic supplementary material.

Supplementary file1 (DOCX 154 kb)

## Data Availability

The data underlying this article cannot be shared publicly due to legal and institutional restrictions and policies. The data that support the findings of this study are available upon reasonable request to the corresponding author.
