# Supplementary material for: Prognostic utility of a multi-biomarker panel in patients with suspected myocardial infarction
Source: Clin Res Cardiol. 2023 Dec 11;113(12):1682–91. doi: 10.1007/s00392-023-02345-7 (PMC11579167; doi:10.1007/s00392-023-02345-7)
Supplement: Supplementary file 1 — Supplementary file1 (DOCX 154 kb) [file 392_2023_2345_MOESM1_ESM.docx]

**Supplementary Material**

**Prognostic Utility of a Multi-biomarker Panel in Patients with Suspected Myocardial Infarction**

***Clinical Research in Cardiology***

Betül Toprak,^a,b^ Jessica Weimann,^a^ Jonas Lehmacher,^a^ Paul M. Haller,^a,b^ Tau S. Hartikainen,^c^ Alina Schock,^a^ Mahir Karakas,^b,d^ Thomas Renné,^e,f,g^ Tanja Zeller,^a,b,h^ Raphael Twerenbold,^a,b,h^ Nils A. Sörensen,^a,b^ Dirk Westermann,^c^ Johannes T. Neumann,^a,b,i^.

^a^Department of Cardiology, University Heart & Vascular Center Hamburg, University Medical Center Hamburg-Eppendorf, Hamburg, Germany; ^b^German Center for Cardiovascular Research (DZHK), Partner Site Hamburg/Kiel/Lübeck, Hamburg, Germany; ^c^Department of Cardiology and Angiology, University Heart Center, Faculty of Medicine, Medical Center, University of Freiburg; ^d^Department of Intensive Care Medicine, Center for Anesthesiology and Intensive Care Medicine, University Medical Center Hamburg-Eppendorf, Hamburg, Germany; ^e^Institute of Clinical Chemistry and Laboratory Medicine, University Medical Center Hamburg-Eppendorf, Hamburg, Germany; ^f^Irish Centre for Vascular Biology, School of Pharmacy and Biomolecular Sciences, Royal College of Surgeons in Ireland, Dublin, Ireland; ^g^Center for Thrombosis and Hemostasis (CTH), Johannes Gutenberg University Medical Center, Mainz, Germany; ^h^University Center of Cardiovascular Science (UCCS), University Heart and Vascular Center Hamburg, Germany; ^i^Department of Epidemiology and Preventive Medicine, School of Public Health and Preventive Medicine, Monash University, Melbourne.

Corresponding author: [j.neumann@uke.de](mailto:j.neumann@uke.de)

**Supplemental Tables**

Online Resource 1 Baseline Levels of Biomarkers in the Multibiomarker Panel 2

Online Resource 2 Follow-up Events 3

Online Resource 3 Univariable Cox Regression Models for 1-year MACE 4

Online Resource 4 Reclassification Tables for Expanded Models 6

**Supplemental Figures**

Online Resource 5 Kaplan-Meier curves for 1-year MACE by Quartiles of

Selected Biomarkers 7

**Supplemental Tables**

**Online Resource 1** Baseline Levels of Biomarkers in the Multi-biomarker Panel

|  | All (N=748) |
| --- | --- |
| Log adiponectin (µg/mL) | 1.7 (1.3, 2.2) |
| Log A2Macro (mg/mL) | 0.7 (0.5, 1.0) |
| Log Apo A-I (mg/mL) | 1.0 (0.8, 1.2) |
| Log Apo A-II (ng/mL) | 5.8 (5.6, 6.0) |
| Log Apo C-I (ng/mL) | 6.0 (5.8, 6.3) |
| Log Apo C-III (µg/mL) | 6.0 (5.7, 6.2) |
| Log Apo H (µg/mL) | 6.1 (5.9, 6.3) |
| Log EN-RAGE (ng/mL) | 4.2 (3.6, 5.1) |
| Log FABP (ng/mL) | 2.3 (2.3, 2.6) |
| Log ferritin (ng/mL) | 5.2 (4.6, 5.9) |
| Log KIM-1 (ng/mL) | -3.1 (-3.1, -2.7) |
| Log LOX-1 (ng/mL) | -0.7 (-0.7, -0.3) |
| Log midkine (ng/mL) | 2.8 (2.3, 3.4) |
| Log myoglobin (ng/mL) | 3.7 (3.3, 4.2) |
| Log NT proBNP (pg/mL) | 6.8 (5.5, 8.1) |
| Log osteopontin (ng/mL) | 3.4 (3.1, 3.7) |
| Log PAI-1 (ng/mL) | 4.1 (3.7, 4.5) |
| Log PARC (ng/mL) | 4.9 (4.5, 5.3) |
| Log RANTES (ng/mL) | 2.5 (2.0, 2.9) |
| Log THP (µg/mL) | -3.2 (-3.6, -2.9) |
| Log TM (ng/mL) | 1.5 (1.3, 1.7) |
| Log TIMP-1 (ng/mL) | 4.5 (4.3, 4.8) |
| Log TTR (mg/dL) | 3.7 (3.5, 3.9) |
| Log TNFR2 (ng/mL) | 1.9 (1.6, 2.3) |
| Log VCAM-1 (ng/mL) | 6.6 (6.4, 6.8) |
| Log copeptin (pmol/L) | 1.9 (1.2, 3.0) |
| Log suPAR (ng/mL) | 1.3 (0.9, 1.7) |
| Log hs cTnI (ng/L) | 1.9 (1.1, 2.9) |
| Log CRP (mg/L) | 1.6 (1.6, 1.9) |
| Values are presented as median (interquartile range).  *Abbreviations:* A2Macro = alpha-2-macroglobulin; Apo = apolipoprotein; CRP = C-reactive protein; EN-RAGE = extracellular newly identified receptor for advanced glycation end-products binding protein; FABP = fatty acid–binding protein; hs-cTnI = high-sensitivity cardiac troponin I; KIM-1 = kidney injury molecule–1; LOX-1 = lectin-like oxidized low-density lipoprotein receptor 1; NT-proBNP = N-terminal prohormone of brain natriuretic peptide; PAI-1 = plasminogen activator inhibitor 1; PARC = pulmonary and activation-regulated chemokine; RANTES = T cell–specific protein RANTES; suPAR = soluble urokinase-type plasminogen activator receptor; THP = Tamm-Horsfall urinary glycoprotein; TIMP-1 = tissue inhibitor ofmetalloproteinases 1; TM = thrombomodulin; TNFR2 = tumor necrosis factor receptor 2; TTR = transthyretin; VCAM-1 = vascular cell adhesion molecule–1. | |

**Online Resource 2** Follow-up Events

|  | **Median follow-up time (95% CI), years** | **Maximal follow-up, years** | **Number of events at 1 year** | **Event rate (%) at 1 year** |
| --- | --- | --- | --- | --- |
| Death | 5.77 (5.71, 5.88) | 6.98 | 40 | 5.35 |
| MACE | 5.79 (5.7, 5.92) | 6.98 | 160 | 22.46 |
| Incident non-fatal MI | 5.64 (5.6, 5.68) | 6.98 | 9 | 1.24 |
| Cardiovascular death | 5.68 (5.66, 5.75) | 6.98 | 15 | 2.16 |
| Revascularization | 5.66 (5.62, 5.7) | 6.98 | 38 | 5.16 |
| Cardiac rehospitalization | 5.7 (5.66, 5.81) | 6.98 | 152 | 20.79 |
| *Abbreviations:* CI = confidence interval; MACE = Major Adverse Cardiovascular Events; MI = myocardial infarction. | | | | |

**Online Resource 3** Univariable Cox Regression Models for 1-year MACE

|  | **Model 1:**  **HR per SD**  **(95% CI)** | **Model 1:**  **p-value** | **Model 2:**  **HR per SD**  **(95% CI)** | **Model 2:**  **p-value** |
| --- | --- | --- | --- | --- |
| Log THP | 0.73 (0.65, 0.84) | <0.0001 | 0.79 (0.69, 0.91) | 0.0015 |
| Log Apo A-I | 0.77 (0.67, 0.88) | 0.00033 | 0.79 (0.69, 0.92) | 0.0025 |
| Log Apo A-II | 0.83 (0.72, 0.96) | 0.014 | 0.9 (0.78, 1.04) | 0.15 |
| Log TTR | 0.83 (0.73, 0.96) | 0.013 | 0.9 (0.78, 1.05) | 0.16 |
| Log Apo C-I | 0.88 (0.75, 1.02) | 0.095 | 0.92 (0.79, 1.08) | 0.32 |
| Log EN-RAGE | 0.99 (0.84, 1.15) | 0.85 | 0.96 (0.81, 1.12) | 0.58 |
| Log RANTES | 1 (0.86, 1.18) | 0.95 | 0.99 (0.85, 1.16) | 0.87 |
| Log Apo C-III | 1.01 (0.86, 1.18) | 0.90 | 1 (0.86, 1.18) | 0.98 |
| Log Apo H | 1.04 (0.89, 1.22) | 0.67 | 1.03 (0.88, 1.2) | 0.70 |
| Log PAI-1 | 1.04 (0.89, 1.22) | 0.58 | 1.03 (0.88, 1.2) | 0.75 |
| Log FRTN | 1.07 (0.92, 1.26) | 0.37 | 1.03 (0.88, 1.21) | 0.69 |
| Log Adiponectin | 1.16 (1, 1.36) | 0.057 | 1.08 (0.9, 1.3) | 0.39 |
| Log LOX-1 | 1.2 (1.06, 1.33) | 0.0052 | 1.12 (0.94, 1.33) | 0.21 |
| Log A2Macro | 1.24 (1.07, 1.44) | 0.0056 | 1.13 (0.96, 1.33) | 0.14 |
| Log CRP | 1.24 (1.09, 1.39) | 0.0019 | 1.15 (1.01, 1.29) | 0.035 |
| Log FABP, heart | 1.27 (1.13, 1.41) | 0.00012 | 1.15 (1.01, 1.31) | 0.041 |
| Log Copeptin | 1.3 (1.12, 1.5) | 0.00059 | 1.17 (1, 1.37) | 0.050 |
| Log Myoglobin | 1.3 (1.13, 1.47) | 0.00023 | 1.2 (1.01, 1.41) | 0.033 |
| Log Midkine | 1.3 (1.13, 1.5) | 0.00038 | 1.2 (1.02, 1.38) | 0.024 |
| Log TM | 1.31 (1.14, 1.49) | 0.00025 | 1.2 (1.03, 1.39) | 0.022 |
| Log hs-TnI 0h | 1.33 (1.17, 1.5) | <0.0001 | 1.21 (1.03, 1.4) | 0.020 |
| Log TIMP-1 | 1.35 (1.21, 1.49) | <0.0001 | 1.22 (1.07, 1.37) | 0.0035 |
| Log PARC | 1.36 (1.17, 1.57) | <0.0001 | 1.24 (1.09, 1.4) | 0.0021 |
| Log VCAM-1 | 1.36 (1.18, 1.54) | <0.0001 | 1.26 (1.1, 1.43) | 0.0015 |
| Log KIM-1 | 1.37 (1.21, 1.53) | <0.0001 | 1.27 (1.1, 1.45) | 0.0019 |
| Log TNFR2 | 1.39 (1.22, 1.56) | <0.0001 | 1.28 (1.12, 1.44) | 0.00038 |
| Log suPAR | 1.43 (1.23, 1.65) | <0.0001 | 1.32 (1.1, 1.55) | 0.0026 |
| Log Osteopontin | 1.5 (1.29, 1.74) | <0.0001 | 1.34 (1.13, 1.6) | 0.0011 |
| Log NT proBNP | 1.81 (1.55, 2.11) | <0.0001 | 1.74 (1.44, 2.09) | <0.0001 |
| **Model 1**: unadjusted/crude. **Model 2**: adjusted for age and sex.  *Abbreviations:* A2Macro = alpha-2-macroglobulin; Apo = apolipoprotein; CI = confidence interval; CRP = C-reactive protein; EN-RAGE = extracellular newly identified receptor for advanced glycation end-products binding protein; FABP = fatty acid–binding protein; HR = hazard ratio; hs-cTnI = high-sensitivity cardiac troponin I; KIM-1 = kidney injury molecule–1; LOX-1 = lectin-like oxidized low-density lipoprotein receptor 1; NT-proBNP = N-terminal prohormone of brain natriuretic peptide; PAI-1 = plasminogen activator inhibitor 1; PARC = pulmonary and activation-regulated chemokine; RANTES = T cell–specific protein RANTES; SD = standard deviation; suPAR = soluble urokinase-type plasminogen activator receptor; THP = Tamm-Horsfall urinary glycoprotein; TIMP-1 = tissue inhibitor ofmetalloproteinases 1; TM = thrombomodulin; TNFR2 = tumor necrosis factor receptor 2; TTR = transthyretin; VCAM-1 = vascular cell adhesion molecule–1. | | | | |

**Online Resource 4** Reclassification Tables for Expanded Models.

| **Clinical model + LASSO-selected biomarkers (Apo A-I, KIM-1, NT-proBNP)** | | | | |
| --- | --- | --- | --- | --- |
|  | Reclassified up, n (%) | Reclassified down, n (%) | NRI | 95% CI |
| Cases | 85 (54.8) | 70 (45.2) | 0.10 | (-0.055, 0.26) |
| Non-cases | 177 (33.8) | 346 (66.2) | 0.31 | (0.23, 0.39) |
| Overall |  |  | 0.41 | (0.24, 0.60) |
| **GRACE Score + LASSO-selected biomarkers (Apo A-I, KIM-1, NT-proBNP)** | | | | |
|  | Reclassified up, n (%) | Reclassified down, n (%) | NRI | 95% CI |
| Cases | 87 (54.4) | 73 (45.6) | 0.088 | (-0.070, 0.23) |
| Non-cases | 199 (36.9) | 340 (63.1) | 0.26 | (0.17, 0.34) |
| Overall |  |  | 0.35 | (0.17, 0.52) |
| *Abbreviations*: Apo = apolipoprotein; CI = confidence interval; GRACE = Global Registry of Acute Coronary Events; KIM-1 = kindey injury molecule-1; NRI = net reclassification index; NT-proBNP = N-terminal prohormone of brain natriuretic peptide. | | | | |

**Supplemental Figures**

**Online Resource 5**  Kaplan-Meier curves for 1-year MACE by Quartiles of Selected Biomarkers.


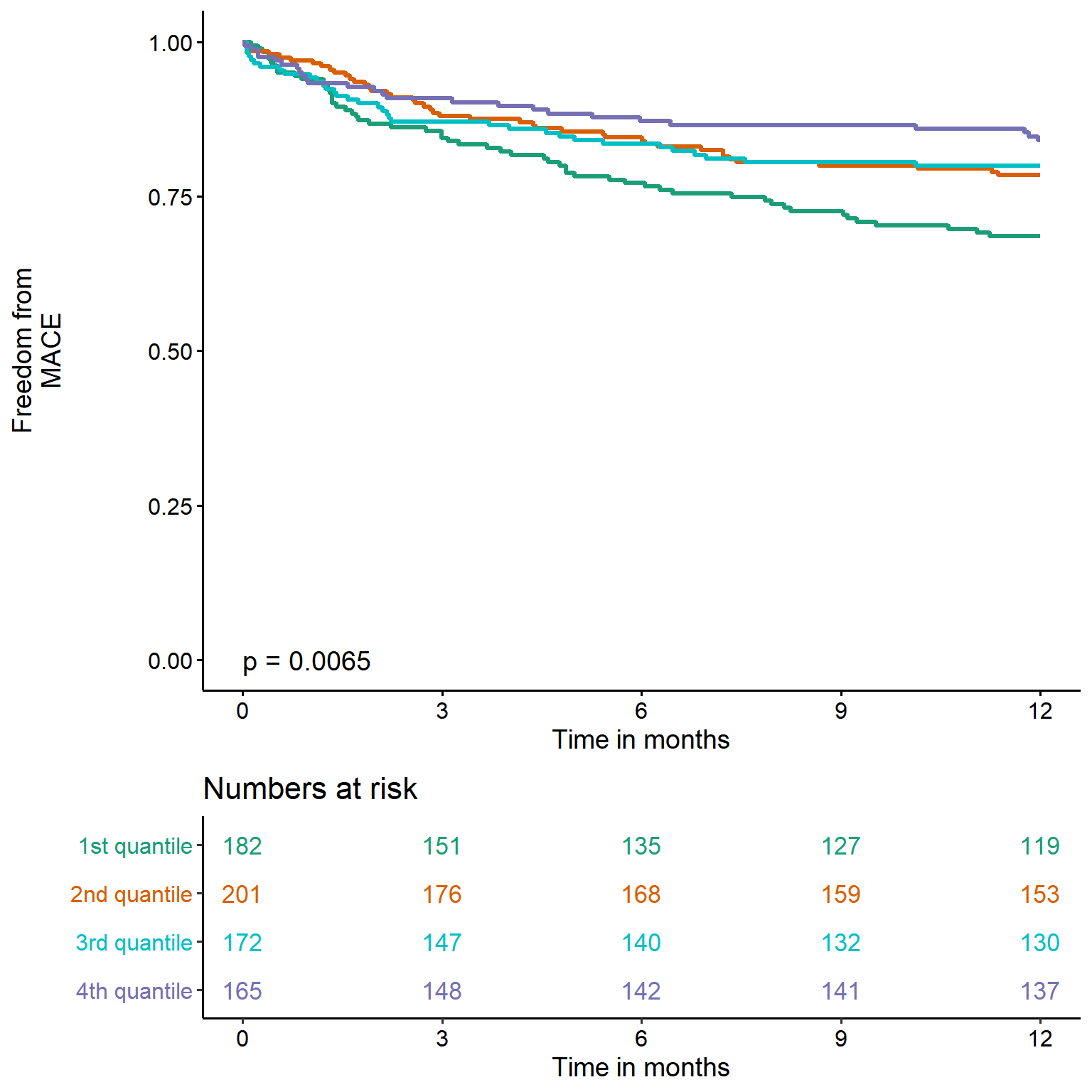


1. Apo A-I


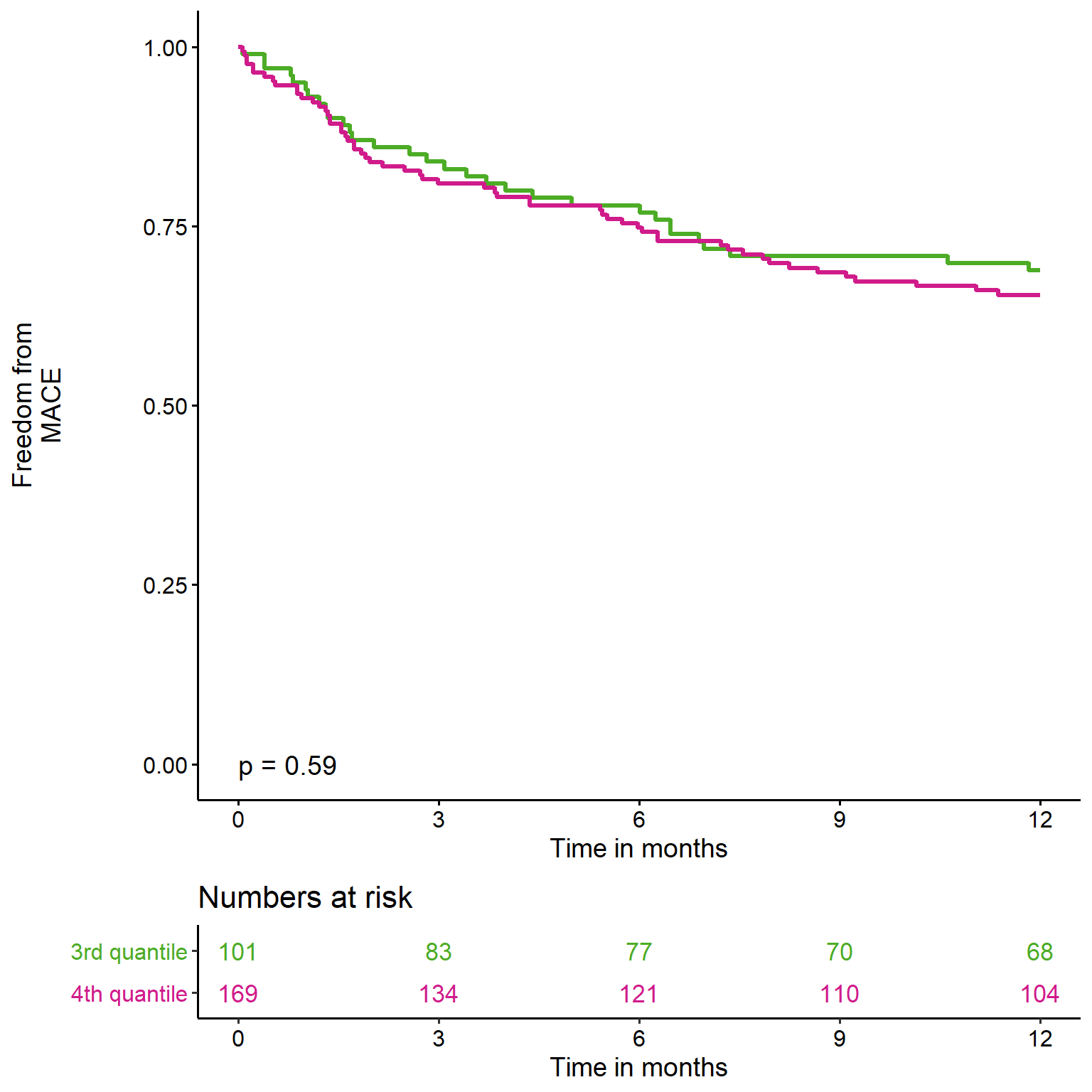


1. KIM-1


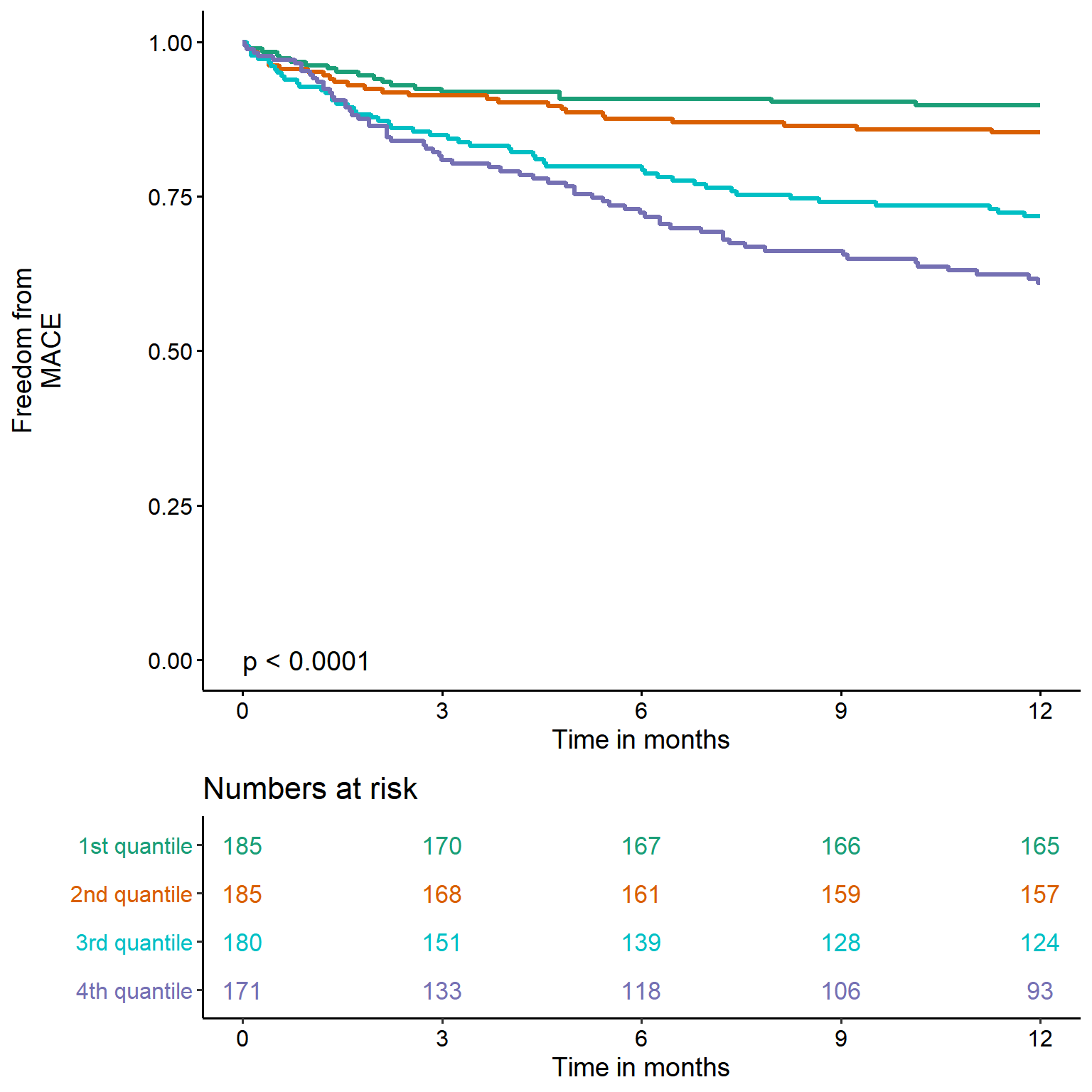


1. NT-proBNP

If lower quantile thresholds are identical, only higher quantiles are shown.

*Abbreviations*: Apo = apolipoprotein; KIM-1 = kindey injury molecule-1; MACE = Major Adverse Cardiovascular Events; NT-proBNP = N-terminal prohormone of brain natriuretic peptide
